# Small lymphocytic lymphoma in true trilineage hematopoietic tissue within heterotopic ossification in an enucleated blind painful eye: a case report

**DOI:** 10.1186/s13256-020-02430-9

**Published:** 2020-07-08

**Authors:** Alfredo Borgia, Sofia Manara, Monica Balzarotti, Paolo Vinciguerra, Alessandra Di Maria

**Affiliations:** 1 Humanitas University, Department of Biomedical Sciences, Via Rita Levi Montalcini 4, 20090 Pieve Emanuele – Milan, Italy; 2 Humanitas Clinical and Research Center – IRCCS, Via Manzoni 56, 20089 Rozzano (Mi), Italy

**Keywords:** Intraocular heterotopic ossification, Extramedullary hematopoiesis, Small lymphocytic lymphoma

## Abstract

**Background:**

The finding of hematological malignancies within bone marrow in heterotopic ossification has been reported only a handful of times previously in the literature. We described a case of true trilineage hematopoiesis in an excised area of heterotopic ossification from an enucleated blind painful eye.

**Case presentation:**

A 70-year-old Caucasian man, positive for asymptomatic lymphoplasmacytic lymphoma, presented with a blind painful right eye in our ophthalmology department to evaluate enucleation bulbi. After enucleation, a histopathologic examination revealed the presence of intertrabecular infiltration in the metaplastic bone marrow of non-Hodgkin B lymphoma, with small lymphocytes, with similar characteristics to the already known disease.

**Conclusion:**

This uncommon case reveals the possibility of the localization of malignant cells within bone metaplasia of intraocular ossification in an enucleated blind painful eye. From a practical point of view, a careful systematic histopathological examination of all resected tissues in patients with a history of malignant neoplastic pathology is necessary to confirm the diagnosis and exclude occult malignancies.

## Background

The presence of bone marrow within heterotopic ossification (HO) has been reported only a few times in the literature, and reports of finding hematological malignancies within the same bone marrow are even rarer. This uncommon case reveals the possibility of localization of malignant cells within bone metaplasia and we want to highlight the importance of a systematic histopathological examination of all resected tissue in patients with a history of malignant neoplastic pathology.

## Case presentation

We report a case of true trilineage hematopoiesis in an excised area of HO from an enucleated blind painful eye.

A 70-year-old Caucasian man presented with a blind painful right eye to our ophthalmology department to evaluate enucleation bulbi. He had a history of a blunt trauma injury to his right eye that occurred 49 years ago, and he subsequently experienced hyphema, vitreous hemorrhages, and traumatic cataract. Over the past 10 years, he experienced episodes of ocular pain phthisis bulbi associated with trigeminal neuralgia. A slit lamp examination of his right eye showed band keratopathy and seclusion pupillae, with a brunescent traumatic cataract. A funduscopic examination of his right eye was not executable due to media opacity. His left eye was normal without any kind of lesions and had an uncorrected visual acuity (UCVA) of 20/20. A right eye ultrasound B-scan examination showed a phthisic eye, with complete retinal detachment, and hyperechogenic tissues with an acoustic shadowing due to calcification projected into the orbital tissue. For that reason, the clinicians decided to perform the enucleation of his right eye. His past medical history was positive for asymptomatic lymphoplasmacytic lymphoma associated with immunoglobulin M (IgM) monoclonal peak diagnosed 12 years before enucleation. At the onset, neither lymphadenopathies nor hepatosplenomegaly were described, and 20% bone marrow infiltration was documented. Our patient was periodically followed up without any treatment with evidence of a slow progressive disease, which did not require treatment up until now. Prior to the enucleation, magnetic resonance imaging (MRI) of his brain was performed to study a possible trigeminal neuralgia and was normal without any cerebral lesions.

**Fig. 1:**
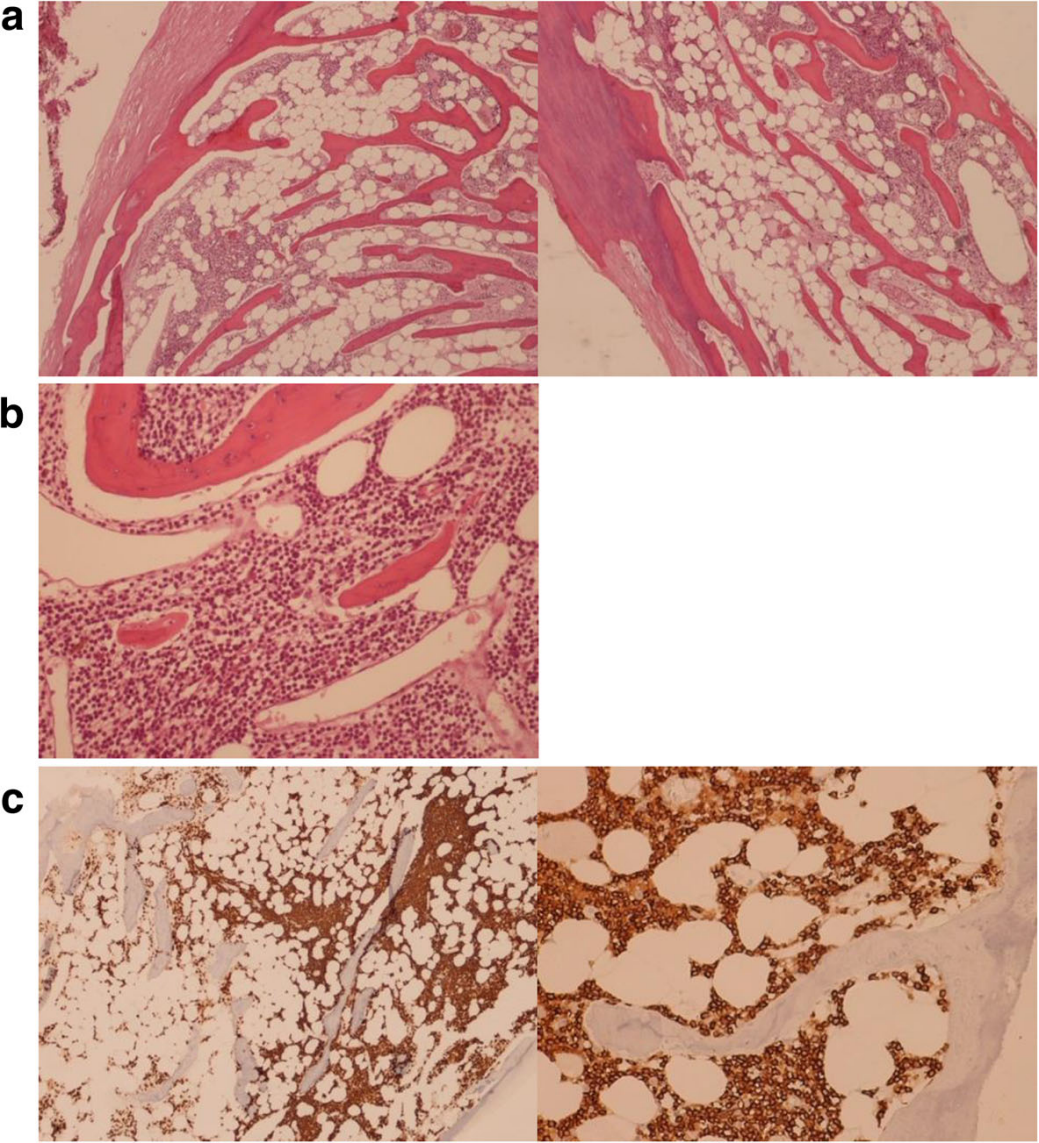
**a** Calcified and ossified sclera flap, with bone trabeculae and intertrabecular spaces inhabited by adipose and hematopoietic medullary elements. There are areas more densely cellulated and occupied by small round cells with little lymphoid-looking cytoplasm (EE= Hematoxylin and Eosin (H&E) × 10 after decalcification treatment). **b** Homogeneous, small-sized lymphoid cells occupying the entire intertrabecular space (EE= Hematoxylin and Eosin (H&E) × 40 after descaling treatment). **c** Immunohistochemical staining with CD20 demonstrates the B lymphoid differentiation of the neoplastic population, occupying the medullary intertrabecular spaces of the newly formed metaplastic bone

The eyeball was removed and marked fibrosis with places of osseous metamorphism was noticed macroscopically. A histopathologic examination revealed diffusive fibrotic and calcified uveo-scleral flaps, with widespread bone metaplasia with hematopoietic marrow, mixed with retinal fragments with atrophic areas alternating with hyperplastic aspects and blood clots (Fig. 1a).

A further typing showed the presence of intertrabecular infiltration in the metaplastic bone marrow of non-Hodgkin B lymphoma, with small lymphocytes, with similar characteristics to the already known disease. Immunostaining was positive for CD20, CD3, CD10, CD43, CD23, CD138, S100 GFAP, MelanA, and Ki-67 (Fig. 1b-c).

## Discussion

Heterotopic bone formation, also known as HO, is the unusual growth of bone outside the skeleton [1]. Heterotopic secondary ossification is usually seen in structures, such as muscles and tissues adjacent to the bone, after musculoskeletal injury or central nervous system or spinal cord trauma [1]. Less commonly encountered sites of heterotopic secondary ossification are in the mesentery, gastrointestinal tract, abdominal incisions and wounds, the walls of blood vessels, the kidneys, the uterus, and the eye [2, 3].

Intraocular ossification is a rare type of metaplasia that can occur after long-standing retinal detachment, some intraocular tumors, chronic inflammation, or eyeball trauma [4]. The incidence of intraocular ossification described in the literature of studies performed on enucleated eyes is up to 18%; however, there are unpublished studies that report an occurrence rate higher than 38% [5, 6]. This condition tends to develop very slowly. Choroidal ossification could be histopathologically traced approximately a year after a traumatic event, whereas it needs 10–20 years to be radiologically identifiable [7, 8].

The pathogenic mechanism underlying intraocular ossification is not completely understood [5, 6, 9]. It seems that the base of intraocular bone formation is metaplastic retinal pigment epithelium (RPE) cells, which after a traumatic and inflammatory stimulus undergo osteoblastic and fibroblastic differentiation. This hypothesis is supported by the frequent observation of islands of RPE cells trapped in the newly formed bone [10].

The presence of bone marrow within HO has been reported only a handful of times in the literature; moreover, the finding of hematological malignancies within the same marrow has been described a few times, as in the case of the formation of myeloma found inside a heterotopic bone marrow in the aortic wall, and in bone metaplasia of a surgical scar [11–13]. This is possibly the first description of true trilineage hematological malignancy in intraocular HO in the literature.

Small lymphocytic lymphoma (SLL) is a non-Hodgkin lymphoma affecting the B lymphocytes of the immune system [14]. SLL often involves the bone marrow, which is why cancer cells have been found within intraocular ossification containing true hematopoietic tissue [15].

## Conclusions

This rare case reveals the possibility of localization of malignant cells in true trilineage hematopoietic tissue within HO in an enucleated blind painful eye. In managing a painful blind eye, we must subject all enucleated specimens to a systematic histopathological examination, to confirm the diagnosis and exclude a hidden intraocular malignancy.

## Data Availability

All data supporting our findings are provided in the manuscript.

## Abbreviations

SLL: Small lymphocytic lymphoma
HO: Heterotopic ossification
RPE: Retinal pigment epithelium

## References

[1] Haque S, Eisen RN, West AB, Shehab D, Elgazzar AH, Collier BD. Heterotopic ossification. J Nucl Med. 2002;43(3):346–53. Available from: http://www.ncbi.nlm.nih.gov/pubmed/8757473. Cited 9 Feb 2020. 11884494

[2] Reardon MJ, Tillou A, Mody DR, Reardon PR. Heterotopic calcification in abdominal wounds. Am J Surg. 1997;173(2):145–7. 10.1016/S0002-9610(96)00415-1 9074383

[3] Haque S, Eisen RN, West A. Heterotopic bone formation in the gastrointestinal tract. Arch Pathol Lab Med. 1996;120(7):666–70. 8757473

[4] Duke-Elder S. Disease of the uveal tract. In: Diseases of the uveal tract System of ophthalmology vol 9; 1966. p. 740–7. Available from: https://ci.nii.ac.jp/naid/10022618406/. Cited 9 Feb 2020.

[5] Vemuganti GK, Honavar SG, Jalali S. Intraocular osseous metaplasia. A clinico-pathological study. Indian J Ophthalmol. 2002;50(3):183. 12355691

[6] Finkelstein EM, Boniuk M. Intraocular ossification and hematopoiesis. Am J Ophthalmol. 1969;68(4):683–90. Available from: http://www.ncbi.nlm.nih.gov/pubmed/4981081. Cited 9 Feb 2020. 10.1016/0002-9394(69)91253-7 4981081

[7] Zografos L, Uffer S, Girard-Othein BCh. Tumeurs osseoses de la choroide. In: Zografos L (ed). Tumeurs intraoculaires. Paris: Masson; 2002. p. 335–50.

[8] Munteanu M, Munteanu G, Giuri S, Zolog I, Motoc AGM. Ossification of the choroid: three clinical cases and literature review of the pathogenesis of intraocular ossification. Rom J Morphol Embryol. 2013;54(3):871–7. Available from: http://www.rjme.ro/. Cited 24 May 2020. 24322043

[9] Lawson BM, Reddy SG, Jody NM. Extensive intraocular osseous metaplasia with bone marrow formation. JAMA Ophthalmol. 2018;136(8):e182377 10.1001/jamaophthalmol.2018.2377. 30098184

[10] Yoon YD, Aaberg TM, Wojno TH, Grossniklaus HE. Osseous metaplasia in proliferative vitreoretinopathy. Am J Ophthalmol. 1998;125(4):558–9. Available from: http://www.ncbi.nlm.nih.gov/pubmed/9559746. Cited 9 Feb 2020. 10.1016/s0002-9394(99)80201-3 9559746

[11] Elliott K, Fitzsimons DW, editors. Ciba Foundation Symposium 11 - Hard Tissue Growth, Repair and Remineralization. Chichester: Wiley; 1973. p. 169–79. (Novartis Foundation Symposia; vol. 11). Available from: http://doi.wiley.com/10.1002/9780470719947. Cited 24 May 2020.

[12] Toyran S, Lin AY, Edward DP. Expression of growth differentiation factor-5 and bone morphogenic protein-7 in intraocular osseous metaplasia. Br J Ophthalmol. 2005;89:885–90. Available from: http://bjo.bmj.com/. Cited 9 Feb 2020. 10.1136/bjo.2004.056374 PMC1772725 15965172

[13] Udoji WC, Krohn NJ. Myelofibrosis and myeloma in heterotopic bone marrow. Arch Pathol Lab Med. 1979;103(6):315–6. Available from: http://www.ncbi.nlm.nih.gov/pubmed/582271. Cited 9 Feb 2020. 582271

[14] Zelenetz AD, Gordon LI, Wierda WG, Abramson JS, Advani RH, Andreadis CB, *et al.* Chronic lymphocytic leukemia/small lymphocytic lymphoma, version 1.2015. J Natl Compr Cancer Netw. 2015;13(3):326–62. 10.6004/jnccn.2015.0045 PMC4841457 25736010

[15] Forest F, Molina L, Stefani L, Peoc’h M. Marginal zone B-cell lymphoma in true trilineage haematopoietic tissue within heterotopic ossification of a surgical scar. J Clin Pathol. 2012;65:1055–6. Available from: http://www.ncbi.nlm.nih.gov/pubmed/22798554. Cited 9 Feb 2020. 10.1136/jclinpath-2011-200633 22798554

